# Influence of Meteorological Parameters on the Prevalence of TEE Detected Left Atrial Appendage Thrombi

**DOI:** 10.3390/diseases12070151

**Published:** 2024-07-12

**Authors:** Franziska Lecker, Klaus Tiemann, Thorsten Lewalter, Clemens Jilek

**Affiliations:** 1 Peter-Osypka-Herzzentrum München, Internistisches Klinikum München Süd, 81379 Munich, Germany; 2 Medical Graduate Center, School of Medicine, Technical University of Munich, 81675 Munich, Germany; 3 Department of Internal Medicine I, University Hospital Rechts der Isar, TUM School of Medicine and Health, Technical University of Munich, 81675 Munich, Germany

**Keywords:** left atrial appendage, thrombus, 3D transesophageal echocardiography, epidemiology, weather, prevention

## Abstract

(1) Background: Meteorological factors seem to exert various effects on human health, influencing the occurrence of diseases such as thromboembolic events and strokes. Low atmospheric pressure in summer may be associated with an increased likelihood of ischemic stroke. The aim of this study was to investigate the potential impact of meteorological conditions on left atrial appendage (LAA) thrombus formation. (2) Methods: A total of 131 patients were included, diagnosed with a first instance of thrombus via 3D transesophageal echocardiography (TEE) between February 2009 and February 2019. Months with frequent thrombus diagnoses of at least 10 thrombi per month were categorized as frequent months (F-months), while months with fewer than 10 thrombus diagnoses per month were labelled as non-frequent months (N-months). The analysis focused on differences in meteorological parameters in two-week and four-week periods before the diagnosis. (3) Results: F-months were predominantly observed in spring and summer (April, May, June, and July), as well as in February and November. During F-months, a higher absolute temperature difference, lower relative humidity, longer daily sunshine duration, and greater wind speed maximum were observed in the two- and four-week periods rather than for N-months. In the two-week period, average temperatures, equivalent temperatures, and temperature maxima were also significantly higher during F-months than N-months. (4) Conclusion: Thrombi in the left atrial appendage are more prevalent during periods characterized by high absolute temperature differences, low relative humidity, and long daily sunshine duration.

## 1. Introduction

Several studies have identified correlations between climate factors and the incidence of deep vein thrombosis, pulmonary artery embolisms, strokes, and myocardial infarction [1,2,3,4,5].

In detail, extreme weather conditions are risk factors of acute coronary syndrome: high outside temperature und long sunshine hours per day in a gender-mixed cohort infarction [6], as well as in a female-only cohort [7], and the quantity and duration of snow fall among men [8].

In analogy to myocardial infraction, ischemic stroke and hospital admission are also associated with high temperature [9,10] or temperature changes [11], whereas hemorrhagic stroke seems not to be affected by extreme temperatures [12].

Stroke and myocardial infarction may be caused by cardiac emboli [13], mainly driven by atrial fibrillation.

Atrial fibrillation (AF) affects approximately 1–2% of the total population and is the most prevalent sustained cardiac arrhythmia in Germany [14]. One significant complication of AF is thromboembolism, such as stroke, primarily attributed to thrombus formation in the left atrial appendage (LAA) [15]. The mortality rate of a stroke is approximately 20%, and the consequences of surviving a stroke are severe [16]: 30% of patients remain disabled, and stroke represents the most common reason for an existing need for long-term care in Germany [16]. Therefore, thromboembolism prophylaxis through the use of anticoagulants or occlusion of the LAA is of decisive importance [17].

The Virchow triad delineates the three fundamental pathomechanisms contributing to thrombus development: alterations in the vessel wall, low blood flow velocities, and shifts in blood composition, leading to hypercoagulability [18]. Inadequate fluid intake or elevated fluid loss during heat waves might result in dehydration, hemoconcentration, and hypercoagulability, subsequently raising the risk of cardiovascular disease and thrombosis [19,20,21].

Until now, the origin and the triggering factors of thrombus formation in the LAA have been poorly understood. We aimed to investigate the influence of meteorological factors on the incidence of LAA thrombus formation.

## 2. Materials and Methods

### 2.1. Study Collective

This retrospective multicenter study encompassed patients diagnosed with an LAA thrombus for the first time by TEE between February 2009 and February 2019 in one hospital and from February 2016 until February 2019 (because of a change in the clinic information system). Those with a pre-existing LAA thrombus or history of recent LAA thrombi were excluded.

Patient data were anonymously gathered from the respective hospital information systems of the two participating institutions: Peter Osypka Herzzentrum at Internistisches Klinikum München Süd GmbH, Munich, Germany; and LAKUMED Kliniken Krankenhaus Landshut-Achdorf, Landshut, Germany.

### 2.2. Transesophageal Echocardiography

First, 2D, biplane 2D, and 3D TEE were performed according to the hospitals’ standard procedures. Multiple planes were acquired in 2D and biplane 2D to identify thrombus formation and to differentiate thrombi from artifacts or pectinate muscles within the LAA. Furthermore, 3D datasets were acquired in all patients. The 2D and 3D datasets were analyzed by two blinded expert readers; in 2 cases, a third reader served to adjudicate the decision. Solid LAA thrombus was defined as a mass visible in at least 2 planes, as confirmed by 3D analysis. Three cases where sludge could not safely be differentiated by offline reading from solid thrombus were not included.

### 2.3. Meteorological Factors

The current location of each patient was used to identify the closest weather station to the home of the patient. For each individual patient, the following weather parameters were collected via the Internet portal of the German Weather Service (DWD) in a time period of 2 and 4 weeks preceding the date of thrombus diagnosis:

- Daily average temperature at 2 m altitude in °C;
- Daily maximum temperature at 2 m altitude in °C;
- Daily minimum temperature at 2 m altitude in °C;
- Daily average relative humidity at 2 m altitude in %;
- Daily average wind speed at 10 m altitude in m/s;
- Daily maximum wind speed at 10 m altitude in m/s;
- Daily sunshine duration in hours;
- Daily precipitation amount in mm;
- Daily mean air pressure at station altitude in hPA (hectoPascal);
- Daily mean water vapor pressure in hPA.

Using the daily data, we calculated the mean values for the two- and four-week periods preceding the initial thrombus diagnosis. The absolute temperature difference was determined by subtracting the temperature minimum from the temperature maximum for the respective two- and four-week intervals. The equivalent temperature was computed using the following formula:

$$Tä[in\;{}^{\circ}C]=t+94341\times \frac{f\times 10^{\frac{7.45\times t}{235+t}}}{p}$$

Tä [in °C] = t + 94,341 × (f ×〖10〗^((7.45 × t)/(235 + t)))/p

where Tä is the equivalent temperature in °Celsius (°C), t is the air temperature in °Celsius (°C), f is the relative humidity in percent (%), and p is the air pressure in hectoPascal (hPA).

Months with an initial diagnosis of at least 10 thrombi were categorized as thrombus-rich months (F-months), while months with fewer than 10 initial thrombus diagnoses per month were labelled as non-frequent diagnosis months (N-months). Meteorological parameters were recorded for the two-week and four-week periods preceding the diagnosis of the thrombus. Subsequently, an analysis was carried out to examine the disparities in meteorological parameters between F- and N-months.

### 2.4. Statistical Analysis

The statistical analysis was conducted using IBM SPSS Statistics (Version 27). The following inferential methods were applied: *t*-test, Mann–Whitney U-test, chi2-test according to Pearson, Fisher exact test, and the correlation coefficients according to Pearson and Spearman. The statistical significance level was set at *p* < 0.05. In multiple testing, the alpha significance level was corrected according to Bonferroni adjustment. To evaluate the significance of the *p*-value, given the different sizes of the two groups, the effect strength was calculated using Pearson’s correlation coefficient, r, in each case. Cross-tabulations and receiver operating-characteristic curve analyses were conducted to differentiate the results from random results.

### 2.5. Ethics

The study was conducted according to paragraph 27 of the Bavarian Hospital Law, which permits retrospective population-based observational research aligned with health services’ research objectives. The special protective measures required for handling data in accordance with paragraph 27 (4,6) of the Bavarian Hospital Law were complied with. Only anonymized data were utilized, and all requirements of the Bavarian Data Protection Law were observed. Consequently, no ethics approval was deemed necessary

## 3. Results

Out of the 131 study patients, 47 (35.9%) were female and 84 (64.1%) were male. The average age of the patient collective at diagnosis was 72.7 years ± 10.1 years (see Table 1). The average age of men at diagnosis was 71.0 years ± 10.8 years, significantly lower than that of women at 75.5 years ± 8.2 years. In total, 99 of the 131 patients were already using anticoagulant medication, with the most common types being sole non-vitamin K antagonist oral anticoagulants (NOACs) (30%), sole antiplatelet agents (16%), or sole Phenprocoumon (13%). Twelve percent of the patients in the study cohort were taking both an NOAC and an antiplatelet agent at the time of thrombus diagnosis. Few patients took antiplatelet agents in conjunction with Phenprocoumon or heparin (5%). Atrial fibrillation was pre-known in 89% of the study’s patients. Two patients with LAA thrombus had no AF or flutter. In the group with pre-existing AF, persistent AF was the most common subtype in 62 patients (53%). In all, 16 patients (14%) had paroxysmal AF, and 13 patients (11%) had permanent AF. In eight patients (7%), the AF was diagnosed together with the thrombus in the LAA and had not been previously recognized.

In total, there were six F- and six N-months identified. F-months were predominantly observed in the spring and summer months of April, May, June, and July, as well as in February and September. January, March, August, September, October, and December were categorized as N-months. In the F-months, a total of 85 thrombi were diagnosed; in the N-months, there were 46 initial diagnoses.

### 3.1. Temperature

The average temperatures in the two-week period before thrombus diagnosis were statistically significantly higher in the F-months than in the N-months (10.8 °C vs. 7.5 °C, *p* = 0.012, r = 0.22). Similarly, significantly higher values in the F-months were observed for temperature maxima (15.8 °C vs. 11.6 °C, *p* = 0.005, r = 0.25), equivalent temperature (27.0 °C vs. 22.5 °C, *p* = 0.034, r = 0.19), and absolute temperature differences (10.0 °C vs. 7.7 °C, *p* < 0.001, r = 0.33) during the two-week period (Table 2). For the four-week period, only the temperature difference showed significantly higher values in the F-months (9.7 °C vs. 8.2 °C, *p* = 0.004, r = 0.25) (Table 3).

In patients not taking antiplatelet or antidiabetic medication within two weeks prior to thrombus diagnosis, there was an association between thrombus formation and high daily mean and maximum temperatures. Similar findings were obtained among patients who were taking antidiabetic medication. Additionally, a positive correlation between temperatures and thrombus formation was noted for patients with permanent or persistent AF two weeks before thrombus diagnosis (mean temp. *p* = 0.006, r = 0.31; max temp. *p* = 0.003, r = 0.34; min temp. *p* = 0.012, r = 0.29) compared to patients with paroxysmal AF. Thrombus formation due to high temperatures was also increased among patients with elevated creatinine values above 1.44 mg/dL (mean temp. *p* = 0.003, r = 0.58; max. temp. *p* = 0.002, r = 0.59; min. temp. *p* = 0.008, r = 0.53) in a period of two weeks before thrombus diagnosis.

### 3.2. Relative Humidity

Relative humidity was significantly lower in the F-months than in the N-months in both the two-week and four-week periods before thrombus diagnosis (*p* < 0.001 in each case, two weeks r = 0.39; four weeks r = 0.33) (Table 2 and Table 3).

### 3.3. Wind Speed

Regarding wind speed, mean wind speed did not differ between F- and N-months in both periods. However, the maximum wind speed was significantly higher in the F-months (Table 2 and Table 3).

### 3.4. Sunshine

Daily sunshine duration was highly significant longer during the F-months in the two-week interval (6.0 h vs. 3.7 h, *p* < 0.001, r = 0.35). For the four-week period, there were significantly longer sunshine hours during the F-months (5.7 h vs. 4.2 h, *p* = 0.004, r = 0.25).

### 3.5. Other Parameters

No differences were found in temperature minimum, precipitation amount, air pressure, and vapor pressure. Furthermore, female gender appeared to be a relevant risk factor for meteorologically dependent thrombus formation, while body weight does not seem to have any influence. For example, compared to the overall collective, women had a higher risk of thrombus formation at high mean wind speeds than men in the four-week periods (women: *p* = 0.034, r = 0.31; men: *p* = 0.497, r = 0.07; overall collective: *p* = 0.075, r = 0.16).

**Figure 1:**
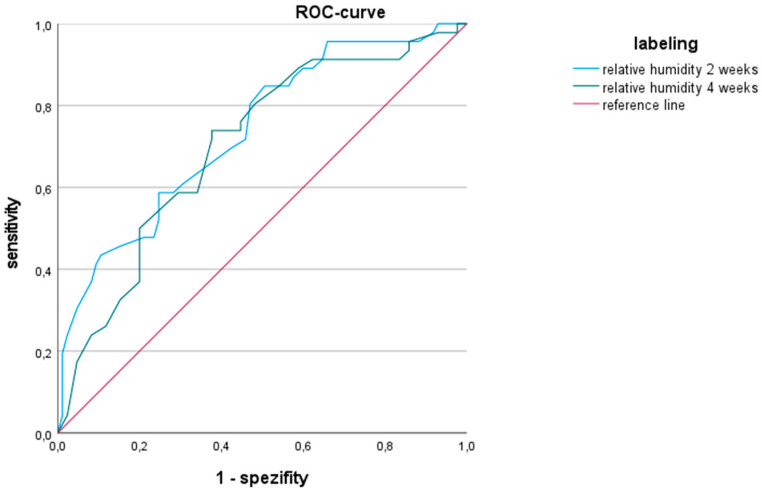
Receiver operating-characteristics curve (ROC) analyzing the predictive power of relative humidity on the probability of thrombus formation in the two- and four-week period. (2 W. Area = 0.735; 95% CI of 0.646–0.824; 4 W. Area = 0.702; 95% CI of 0.609–0.795).

The ROC curve analysis (Figure 1) yielded reliable results for both time periods. In the two-week period (2-week area = 0.735; 95% confidence interval CI of 0.646–0.824), it proves to be a slightly more effective variable for assessing the likelihood of thrombi based on relative humidity than in the four-week period (4-week area 0.702; 95% CI of 0.609–0.795). Accordingly, higher average relative humidity is associated with N-months and is thus indicative of lower thrombus formation.

## 4. Discussion

In the present study, elevated absolute temperature differences, reduced relative humidity, increased maximum wind speed, and prolonged daily sunshine periods indicative of hot spells in both the two-week and four-week periods were identified as risk factors for thrombus formation in the LAA. The majority of thrombi was diagnosed in the spring and summer months of April, May, June, and July, and also in February and November.

Compared to the Gutenberg Health Study (GHS), prospectively collecting data on the health status and its changes in a population cohort since 2007 [14], the collectives are very similar in terms of age, gender distribution, and BMI. However, it is noticeable that the patients with LAA thrombus have a higher prevalence of the disease. Above all, there is a significantly higher prevalence of heart failure and diabetes mellitus, as well as post-apoplexy, compared to the patients in the Gutenberg Health Study with AF. The Prevalence of LAA thrombus was not targeted in the Gutenberg Health Study.

Our findings align with several published studies on stroke incidence. A study from 2019 demonstrated a seasonal increase in the incidence of ischemic strokes compared to hemorrhagic strokes with higher temperature and longer sun exposure [21]. Additionally, concerning air temperature and sunshine, our study highlighted the significant impact of low relative humidity on thrombus formation in the LAA. Our findings are in line with a study from Augsburg on the risk of stroke: dry and hot air masses were associated with an increased risk for macroangiopathic strokes and reduced risk for hemorrhagic strokes, while dry and cold air masses were associated with a higher risk of intracerebral hemorrhage and a lower risk of ischemic stroke [3].

A meta-analysis of 20 studies discovered an association between low air pressure in summer and an increased likelihood of ischemic stroke, along with an escalating risk linked to high relative humidity in summer [2]. Interestingly, there was no direct relationship between air temperature and the incidence of ischemic stroke [2]. A study from Japan by Pan et al. reported an elevated risk of heart failure, ischemic heart disease, and stroke for days with particularly cold temperatures [22]. This aligns with our study, as ischemic heart disease and stroke may originate not only from thromboembolism but also from arteriosclerosis and vasoconstriction, acutely triggered by low temperatures [23]. Heart failure may be an effect of acute ischemic heart disease [24]. For example, one study investigated that inhaling cold air compared to thermoneutral air during exercise increases the degree of regional myocardial ischemia in patients with ischemic heart disease [25].

Referring to the Virchow triad, we pathophysiologically assume a change in the blood composition with subsequent dehydration due to weather changes [18,20]. In both the two- and four-week periods before thrombus diagnosis, the daily sunshine duration was significantly longer, and relative humidity was significantly lower in the F-months compared to the N-months. Significantly higher average temperatures, maximum temperatures, and equivalent temperatures in the F-months were observed only in the two-week period. The absolute temperature differences were the only parameter that showed highly significant values in both periods.

Several studies on the occurrence of myocardial infarctions have also emphasized the critical role of temperature differences, but a meta-analysis showed inconsistent evidence [1]. We are in line with several trials showing a dependency on temperature differences: For example, Amiya et al. showed that high intraday temperature differences play an important role in the development of acute myocardial infarctions [26]. A large study in Canada examined the impact of extreme temperatures on the number of hospitalizations for coronary heart disease (CHD), myocardial infarction, and stroke [27]. The study revealed that, on particularly cold days, the number of hospital admissions due to CHD increased by 9%, due to acute myocardial infarction by 29% and due to strokes by 11% compared to days with optimal temperatures [27]. Even on particularly hot days, the number of CHD admissions rose by 6% [27]. The influence of cold days may be attributed to the pathomechanism of coronary vasoconstriction in ischemic heart disease [28]. Hot days may have an impact on dehydration, change in blood composition, and, as a consequence, thrombus formation.

In summary, the relevance of meteorologically induced occurrences of diseases was confirmed in our work, as well as by previous study results. Several studies describe an increased mortality and morbidity of cardiovascular diseases during high temperatures and heat waves, especially for strokes and CHD [29]. Considering contradictory study results of absolute temperature values, we hypothesize that it could be the temperature differences that predispose for thrombus formation and thromboembolic events.

In light of climate change and anticipated further increases in global temperatures, along with a rise in extreme climatic events such as heat waves, droughts, and storms, medical meteorology will become even more important in the future [30]. The development of early warning systems for patients at risk and the early adoption of prophylactic measures are currently under discussion [31].

### Limitations of the Study

The study’s most relevant limitation is the unknown time of thrombus formation, given that LAA thrombus is not an acute disease, and determining the precise time of formation is impossible by TEE. It cannot be conclusively asserted that the thrombus formed in the two or four weeks prior to initial diagnosis. However, conducting a prospective study with a design involving regular TEE examinations to pinpoint the exact period of thrombus formation is unfeasible, primarily due to ethical considerations.

The effects on laboratory parameters affected by hot temperature and possible dehydration could not be analyzed due to the retrospective design.

A minor limitation is that weather data of the home address were used. There exists a slight uncertainty whether the patient was present at the home address during the weeks before thrombus diagnosis.

## 5. Conclusions

Thrombi in the left atrial appendage are more prevalent during periods characterized by high absolute temperature differences, low relative humidity, and long daily sunshine duration. The susceptibility to thrombus formation in high temperatures is observed in individuals of female gender, those with permanent or persistent atrial fibrillation, and those with elevated creatinine levels.

## Figures and Tables

**Table 1:** Patients’ characteristics.

Patient Characteristics	LAA Thrombus Study (*n* = 131)
Median Age	73 Jahre
Male gender	64%
Average BMI	28 kg/m^2^
Heart failure	62%
Arterial hypertension	77%
Diabetes mellitus	31%
State after apoplexy	16%
Dyslipidemia	37%
Diuretic medication	58%
Antidiabetic medication	18%
Anticoagulant medication	76%
nOAK therapy alone	30%
Antiplatelet agents alone	16%
Coumarin alone	13%
nOAK and antiplatelet agent	12%

**Table 2:** Statistical analysis of the two-week meteorological parameters with representation of the mean values ± standard deviation. Significant *p*-values are highlighted in red.

Meteorological Parameters Average 2 Weeks before Diagnosis	F-Months (85)	N-Months (46)	*p*-Value	Effect Strength, r
Average temperature (°C)	10.8 ± 6.5	7.5 ± 7.3	0.012	0.22
Temperature maximum (°C)	15.8 ± 7.5	11.6 ± 8.5	0.005	0.25
Temperature minimum (°C)	5.9 ± 5.6	3.8 ± 6.4	0.057	0.17
Absolute temperature difference (°C)	10.0 ± 2.8	7.7 ± 2.9	<0.001	0.33
Relative humidity (%)	75 ± 9	83 ± 9	<0.001	0.39
Mean wind speed (m/s)	2.8 ± 0.6	2.8 ± 1.0	0.073	0.16
Maximum wind speed (m/s)	9.5 ± 1.7	8.5 ± 2.3	0.003	0.26
Sunshine duration (h)	6.0 ± 3.0	3.7 ± 2.6	<0.001	0.35
Precipitation amount (mm)	2.0 ± 1.7	2.1 ± 1.6	0.506	0.06
Mean air pressure (hPa)	968 ± 12	966 ± 17	0.845	0.02
Average vapor pressure (hPa)	9.9 ± 3.3	9.3 ± 4.1	0.151	0.13
Equivalent temperature (°C)	27.0 ± 12.0	22.5 ± 14.1	0.034	0.19

**Table 3:** Statistical analysis of the four-week meteorological parameters with representation of the mean values ± standard deviation. Significant *p*-values are highlighted in red.

Meteorological Parameters Average 4 Weeks before Diagnosis	F-Months (85 ED)	N-Months (46 ED)	*p*-Value	Effect Strength, r
Average temperature (°C)	10.1 ± 6.4	8.2 ± 7.3	0.090	0.15
Temperature maximum (°C)	15.1 ± 7.3	12.4 ± 8.5	0.058	0.17
Temperature minimum (°C)	5.3 ± 5.6	4.3 ± 6.3	0.225	0.11
Absolute temperature difference (°C)	9.7 ± 2.6	8.2 ± 2.7	0.004	0.25
Relative humidity (%)	76 ± 9	82 ± 8	<0.001	0.33
Mean wind speed (m/s)	2.8 ±0.5	2.8 ± 0.8	0.075	0.16
Maximum wind speed (m/s)	9.3 ± 1.5	8.7 ± 2.0	0.004	0.25
Sunshine duration (h)	5.7 ± 2.7	4.2 ± 2.6	0.004	0.25
Precipitation amount (mm)	2.0 ± 1.3	1.9 ± 1.1	0.965	<0.001
Mean air pressure (hPa)	968 ± 12	968 ± 14	0.851	0.02
Average vapor pressure (hPa)	9.6 ± 3.1	9.5 ± 4.0	0.472	0.06
Equivalent temperature (°C)	25.8 ± 11.5	23.5 ± 14.0	0.186	0.12

## Data Availability

Data is contained within the article.
